# Role of Organically-Modified Zn-Ti Layered Double Hydroxides in Poly(Butylene Succinate-Co-Adipate) Composites: Enhanced Material Properties and Photodegradation Protection

**DOI:** 10.3390/polym13132181

**Published:** 2021-06-30

**Authors:** Jie-Mao Wang, Hao Wang, Erh-Chiang Chen, Yun-Ju Chen, Tzong-Ming Wu

**Affiliations:** Department of Materials Science and Engineering, National Chung Hsing University, 250 Kuo Kuang Road, Taichung 402, Taiwan; D9866023@mail.nchu.edu.tw (J.-M.W.); eddie115923@icloud.com (H.W.); erchiang.chen@gmail.com (E.-C.C.); bitcoin2243@gmail.com (Y.-J.C.)

**Keywords:** photodegradation, biodegradable polymer, poly(butylene succinate-co-adipate), Zn-Ti LDH

## Abstract

In this research, the effects of Zn-Ti layered double hydroxide (Zn-Ti LDH) as a UV-protection additive, which was added to the poly(butylene succinate-co-adipate) (PBSA) matrix, were investigated. Stearic acid was used to increase the hydrophobicity of Zn-Ti LDH via ion-exchange method. Transmission electron microscopy images of PBSA composites showed that modified Zn-Ti LDH (m-LDH) well-dispersed in the polymer matrix. Due to the effect of heterogeneous nucleation, the crystallization temperature of the composite increased to 52.9 °C, and the accompanying crystallinity increased to 31.0% with the addition of 1 wt% m-LDH. The additional m-LDH into PBSA copolymer matrix significantly enhanced the storage modulus, as compared to pure PBSA. Gel permeation chromatography and Fourier transform infrared spectroscopy analysis confirmed that the addition of m-LDH can reduce the photodegradation of PBSA.

## 1. Introduction

Using biodegradable polymers as substitutes for common plastics has attracted immense interest in reducing the environmental impact of plastic waste [1,2]. Nanomaterial additives is one of the effective methods to enhance the mechanical, thermal, and crystallization properties of biodegradable polymers [3,4]. Many studies have been conducted on the effect of nanomaterials on the degradation behavior of biodegradable polymers in the soil, compost, or simulated environments in the laboratory [5,6,7]. However, the impacts of light, water, and temperature on the degradability of polymers in the natural environment require further investigation. These factors usually deteriorate the properties of biodegradable polymers and affect their lifetime. For polymeric materials that are often exposed to outdoors, sunlight is the main cause of photodegradation and performance loss [8,9]. The photodegradation behavior of several biodegradable polymers, including poly(l-lactide) (PLA), poly(butylene adipate-co-terephthalate) (PBAT), and poly(butylene succinate-co-adipate) (PBSA) have been reported [10,11,12]. The photodegradation feature of the polymer itself and the influence of nanomaterials as fillers have received extensive attention to increase the lifetime of biodegradable polymers under sunlight exposure. Chen et al. prepared a nanocomposite by PBAT and clay, and showed that inorganic particles can absorb or reflect photon energy and reduce the intensity of the light, inhibiting the photodegradation of the polymer [13]. Zhang et al. discussed the effect of ZnO on the photodegradation of PBSA [14] and showed that ZnO hinders the photodegradation of PBSA, but does not significantly change the photodegradation mechanism of PBSA. The ultraviolet light with high energy UV-C (wavelengths 100–280 nm) is absorbed by the earth’s atmosphere while allowing the transmission of UV-B (280–320 nm) and UV-A (320–400 nm) [15]. Therefore, UV-B and UV-A contribute to the photodegradation of most polymers that are used outdoors. To reduce the photodegradation of biodegradable polymers, the UV-B and UV-A absorption capacity of nanomaterials should be further investigated [9,16].

The layered double hydroxide (LDH) conventionally prepared by bivalent and trivalent cations has been well-known for its ability to affect mechanical, crystallization, thermal, and biodegradation properties of biodegradable polymers [17,18]. The general formula for these LDHs is (M^2+^_1−x_M^3+^_x_(OH)_2_)(A^n−^)_x/n_ · mH_2_O, where M^2+^ and M^3+^ are divalent (Mg^2+^, Zn^2+^, Cu^2+^) and trivalent (Al^3+^, Cr^3+^, Fe^3+^) cations, and A^n−^ represents interlamellar anions [19]. The distance between two adjacent layers, which depends mainly on the nature of the interlayer species and their electrostatic interactions with the main layers, can be adjusted by introducing anionic compounds into the interlayer by ion exchange to replace its native anions [20]. The reports also pointed out that by modifying the LDH with a hydrophobic aliphatic carbon chain, the compatibility between originally hydrophilic LDH and the hydrophobic polymer can be improved [21]. The eco-friendliness and biocompatibility of LDHs have been demonstrated [22]. Zn-Ti LDH, which consists of bivalent (Zn^2+^) and tetravalent (Ti^4+^) cations, was developed by Saber et al. [23]. Compared to LDHs with other metal ions (e.g., Mg-Al LDH and Zn-Al LDH), Zn-Ti LDH can provide better protection in broadband UV [24]. Wang et al. also found that Zn-Ti LDH is a safe UV-shielding material as it has lower photocatalytic activity than TiO_2_ and ZnO [15]. Ekambaram et al. also gave same discussion and indicated its ability to shield UV radiation [25]. In the study of Naseem et al., Zn-Ti LDH as a UV-absorbing nanomaterial provided a method for protecting polypropylene from UV-vis degradation [26].

PBSA is an aliphatic biodegradable copolyester, which synthesized via polycondensation of 1,4-butanediol in the presence of succinic and adipic acids [27]. It is worth noting that 1,4-butanediol and succinic acid can not only be extracted from oil, but also via fermentation [28]. The photodegradation reaction of PBSA produces carboxyl end groups (C=O) and chain scission. At the same time, the peak of C=O in the FTIR spectrum can be used to study the evolution of photodegradation. [14,29]. Due to the appropriate degradation rate, thermal stability, mechanical property, and good processability, practical applications of PBSA can be found in mulch films, where the photodegradation stability is a crucial property. The characteristics of Zn-Ti LDH, such as excellent biocompatibility, broadband UV protection, and lower photocatalytic activity, makes it suitable as a UV-protection additive in the PBSA matrix [14,29]. Reviewing past literature regarding PBSA nanocomposites, many studies focus on the addition of nanomaterial and its modification to achieve a better dispersion in the PBSA matrix [5,17,18]. Therefore, to improve the dispersion of Zn-Ti LDHs in PBSA, biocompatible and nontoxic stearic acid (SA) was used to modify in this study [30]. The changes in the physical properties of PBSA composites at various photodegradation periods were investigated. The crystallization, rheology, and thermal and mechanical properties of the PBSA composites were evaluated for their practical applications.

## 2. Materials and Methods

### 2.1. Materials

Commercial PBSA was purchased from Mitsubishi Chemical Co. (Tokyo, Japan), under the trade name BioPBS™ FD92PM. Zinc nitrate hexahydrate (Zn(NO_3_) _2_·6H_2_O), titanium tetrachloride (TiCl_4_), urea (CH₄N₂O), sodium hydroxide (NaOH), and stearic acid (SA, C_18_H_38_O_2_) were acquired from Sigma-Aldrich (St. Louis, MO, USA). All chemicals were used without further purification.

### 2.2. Synthesis and Modification of m-LDH

Zn-Ti LDH was synthesized by the co-precipitation method. TiCl_4_ (0.44 mL), Zn(NO_3_)_2_·6H_2_O (4.76 g) and urea (6.0 g) were dissolved in deionized water, the mixture was stirring at 95 °C for 48 h under nitrogen atmosphere. After preparation, the obtained insoluble (i.e., Zn-Ti LDHs) was washed by deionized water and ethanol, eventually dried at 80 °C in vacuum 24 h. The SA modification of Zn-Ti LDH was used by ion-exchange method. SA (0.2 M) and Zn-Ti LDH (0.5 g) were added into the deionized water. The solution controlled at pH = 10 to 11 by drop 0.1 M aqueous NaOH and stirring at 80 °C for 24 h under nitrogen atmosphere. The produced m-LDH was washed by deionized water and ethanol several times, then dried at 50 °C in vacuum 24 h.

### 2.3. PBSA/LDH Composites Preparation

PBSA was dissolved in dichloromethane (Sigma-Aldrich) for 2 h. Different amount of m-LDH were added into dichloromethane and ultrasonicated for 10 mins to reach dispersions. After that, the m-LDH solution was added into PBSA solution slowly and stirring for 1 h. The mix solution was solvent casting onto a glass petri dish at room temperature for 24 h then dried in vacuum at 50 °C for 48 h. The obtained composites are identified as PBSA/m-LDH-x, where x is the wt% of m-LDH in composite. For further characterization, samples were hot-pressed to plate at 120 °C then cooling at room temperature.

### 2.4. Artificial Photodegradation Test

The samples were irradiated under artificial conditions with an artificial light source (Philips CLEO HPA 400S, Amsterdam, Netherlands), which has a mainly radiation between 300 and 400 nm. The sample dimension is 10 mm × 10 mm × 1 mm (L × W × T) prepared by hot-press. The temperature of sample surface is about 45 °C. The relative humidity of the environment is about 50%. 

### 2.5. Characterization

The TEM images of PBSA composites were performed using JEM-2010 (JEOL, Tokyo, Japan). The scanning electron microscopy (SEM) images of PBSA composites were performed using JSM-6700F (JEOL, Tokyo, Japan). The contact angle was carried out on FTA200 (First Ten Angstroms, Portsmouth, VA, USA). FTIR experiments of Zn-Ti LDH, SA, and m-LDH were carried out on a spectrometer (Spectrum One, Perkin-Elmer, Waltham, MA, USA) in the range from 450 to 4000 cm^−1^. The PBSA and its composites were performed in attenuated total reflection (ATR) mode. The UV absorption spectrum was acquired by a U-3900 UV–vis spectrophotometer (Hitachi, Tokyo, Japan) in the range of wavelength from 250 to 400 nm. X-ray diffractometer (Bruker D8, Karlsruhe, Germany) equipped with a Ni-filtered Cu Kα radiation was used for the experiments of wide-angle X-ray diffraction (WAXD). The measurements of WAXD were carried out in the range of 2θ = 1–40° at scanning rate of 1°/min. The thermal degradation of specimens was operated using thermal gravimetric analyzer (TGA 2950, TA Instruments, New Castle, DE, USA). The experiment was carried out from room temperature to 600 °C under atmospheric environment at a heating rate of 10 °C/min. The crystallization behavior was carried out by a Pyris Diamond DSC (Perkin-Elmer, Waltham, MA, USA) and all experiments were performed under nitrogen environment. All specimens were heated to the designed temperatures (T_ds_), which were about 40 °C higher than the melting temperatures of neat PBSA, at a rate of 10 °C/min and held for 5 min. Then, they were cooled to −30 °C at a rate of 10 °C/min, which was called 1st cooling. Finally the samples were heated to T_ds_ at a rate of 10 °C/min, which was called 2nd heating. The crystallinity degree (X_c_) of neat PBSA and the composites was obtained according to enthalpy of the melting peak (∆H_f_) of 2nd heating. The storage modulus (E′) was evaluated by DMA8000 (Perkin-Elmer, MA, USA) from −80 to 40 °C at 2 °C/min heating rate and 1 Hz constant frequency. Sample size is 20 mm × 10 mm × 0.5 mm (L × W × T). The experiment was performed in atmospheric environment. Molecular weights of the samples were determined by GPC (LC-4000, JASCO, Tokyo, Japan) with a refractive index detector (RI-4030, JASCO, Tokyo, Japan) calibrated with standard polystyrene. Dichloromethane was used as the mobile phase with a 1 mL/min flow rate.

## 3. Results and Discussion

### 3.1. Characterization of m-LDH

**Figure 1:**
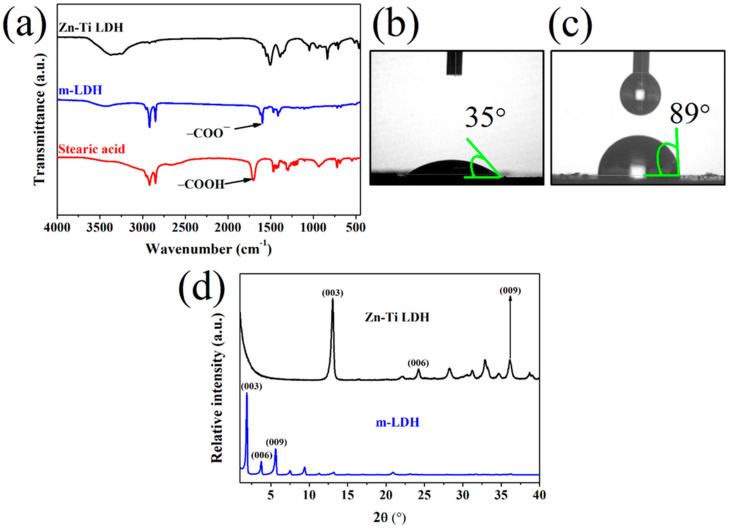
(**a**) The FTIR result of Zn-Ti LDH, m-LDH, and SA; the photographs of water contact angle on the surface of (**b**) Zn-Ti LDH and (**c**) m-LDH; (**d**) XRD result of Zn-Ti LDH and m-LDH.

The FTIR spectra of Zn-Ti LDH, m-LDH, and SA are shown in Figure 1a. A broad absorption band between 3200 and 3400 cm^−1^ of Zn-Ti LDH indicates the stretching mode of hydroxyl groups and physisorbed water in the interlayer of LDH. The bands at 1508, 1388, and 1047 cm^−1^ are attributed to the interlayer CO_3_^2−^. In addition, the bands below 1000 cm^−1^ are metal-oxygen (MO, O-M-O, or M-O-M) signals [15,23,24]. After the chemical modification with SA, the absorption bands at 2920, 2850, and 1462 cm^−1^ are related to the CH_2_ asymmetric vibrations, symmetric vibrations, and scissor mode of SA, respectively [31]. Notably, the bands attributed to the CO_3_^2−^ of Zn-Ti LDH and the band at 1701 cm^−1^ attributed to the COOH group of SA disappeared or became weaker in the spectrum of m-LDH. At the same time, the clear absorption band at 1596 cm^−1^ of m-LDH is due to COO^−^ stretching vibrations, indicating that the SA was converted to stearate anion (from H_3_C(CH_2_)_16_COOH to H_3_C(CH_2_)_16_COO^−^) [23]. Figure 1b,c give the photographs of water droplets on the surface of Zn-Ti LDH and m-LDH, respectively. The wettability of Zn-Ti LDH changed with SA modification. Compared to Zn-Ti LDH, a larger water contact angle of m-LDH means lower hydrophilicity, which may result in a better dispersion in the PBSA matrix.

Figure 1d shows the XRD patterns of Zn-Ti LDH and m-LDH. The diffraction peaks of Zn-Ti LDH at 2θ = 13.05°, 24.33°, 28.23°, 32.90°, 33.28°, 36.14°, and 38.70° correspond to the crystal planes of (003), (006), (012), (100), (101), (009), and (015), respectively [23,32]. The d-spacing of (003), (006), and (009) calculated by Bragg’s law are 0.68, 0.37, and 0.25 nm, respectively. After chemical modification using SA, the diffraction peaks of (003), (006), and (009) planes of LDH shifted to smaller angles at 2θ = 1.88°, 3.73°, and 5.64°, respectively. The d-spacing corresponding to (003), (006), and (009) planes of m-LDH increases to 4.69, 2.37, and 1.57 nm, respectively. The chemical modification can expand the interlayer distance of LDH, indicating that the ion exchange of SA was successful and consistent with the above FTIR results. In addition, the chain length of the stearate anion is about 2.25 nm [23]. From the increase in d-spacing from 0.68 nm of ZN-Ti LDH to 4.69 nm of m-LDH, we deduce that the intercalation of SA formed bilayer structures with an inclined angle of 63°. 

**Figure 2:**
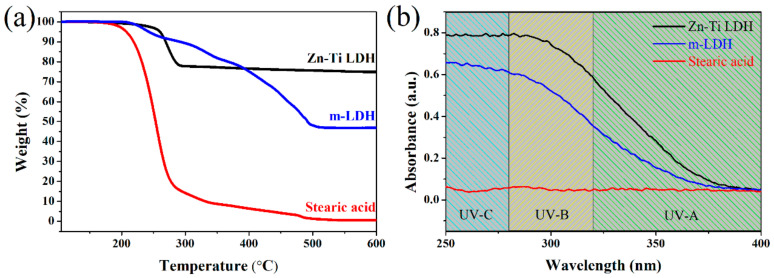
(**a**) TGA and (**b**) UV-vis result of Zn-Ti LDH, m-LDH, and SA.

The TGA curves of Zn-Ti LDH, m-LDH, and SA performed in the air environment are shown in Figure 2a. The 10% weight loss and Char yield weight percent at 600 °C of Zn-Ti LDH, m-LDH, and SA are shown in Table A1. The main thermal decomposition of Zn-Ti LDH occurs from 200 to 300 °C, which corresponded to the evaporation of carbonate anions [23]. After chemical modification using SA, there is no rapidly weight drop as same as Zn-Ti LDH at the temperature below 300 °C, and the residual weight of m-LDH is 46.9% at 600 °C. Combining the analysis results of FTIR, XRD and TGA, it can be seen that SA had been inserted into the Zn-Ti LDH laminates by ion exchange method. It can be seen from the change of contact angle that SA was also adsorbed on the surface of m-LDH.

The UV–vis absorbance spectrum of Zn-Ti LDH, SA, and m-LDH are shown in Figure 2b. An absorption signal from 250 to 370 nm is observed for Zn-Ti LDH, which includes whole UV-B and most of the UV-A irradiation range. Simultaneously, no significant UV-absorbing character of SA is observed from 250 to 400 nm. After modification, the absorption intensity of m-LDH is lower than Zn-Ti LDH. However, the m-LDH still shows a significant absorption signal from 250 to 370 nm, which indicates a good application potential in UV irradiation protecting.

### 3.2. Characterization of PBSA and Its Composites

**Figure 3:**
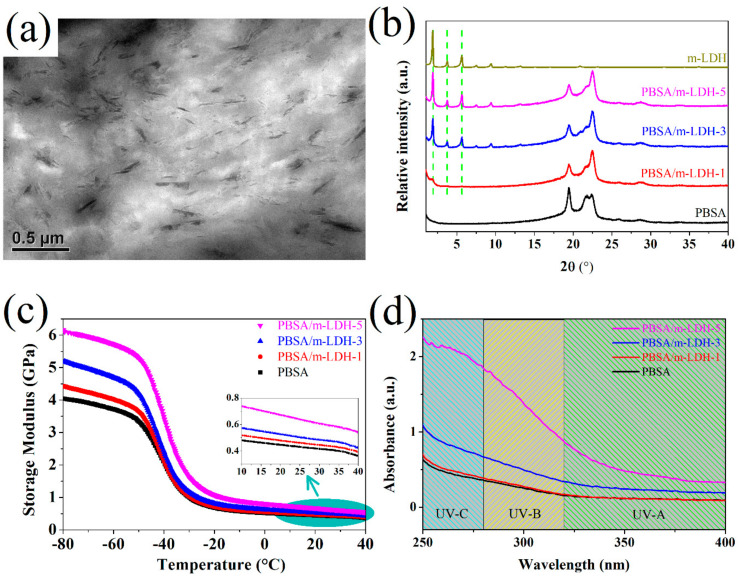
TEM image of (**a**) PBSA/m-LDH-5 and (**b**) XRD, (**c**) DMA, and (**d**) UV-vis result of PBSA and its composites.

Figure 3a shows the TEM images of PBSA/m-LDH-5. It can be seen that the stacking layers of m-LDH dispersed in PBSA matrix. Figure 3b shows the XRD diffraction patterns of PBSA and its composites. The XRD experimental data of PBSA shows three diffraction peaks at 2θ = 19.4°, 21.7°, and 22.4°, corresponding to (020), (021), and (110) crystallographic planes of monoclinic PBS, respectively [17,18]. The XRD results show that the addition of m-LDH did not change the crystal structure of PBSA. In addition, by increasing m-LDH content, the peak positions of (003), (006), and (009) planes for m-LDH remained unchanged, and the intensities of these peaks increased. These results indicate that the initial layer stacking structure of m-LDH is still present in PBSA.

The mechanical properties, represented by the storage modulus (E′), of the PBSA and its composites evaluated by DMA are shown in Figure 3c. The phenomenon of E’ dropping rapidly at approximately −40 °C contributes to the occurrence of glass transition of PBSA [27]. The effect of m-LDH on the mechanical properties of PBSA can be seen from E′ at the temperature below T_g_. The value of E′ at −80 °C increases significantly with increasing filler content. Detailed data are presented in Table 1. The enhancement of mechanical properties for the PBSA composites may be attributed to the reinforcement effect of the additional stiffness of LDH [5]. In addition, the PBSA/m-LDH-5 composites exhibit the highest mechanical properties at 25 °C, as shown in Figure 3c and Table 1.

The UV–vis absorbance spectrum of PBSA and its composites are shown in Figure 3d. A relatively weak absorption of PBSA was observed in the range from 250 to 320 nm, which is attributed to the absorption of the carbonyl group [14]. By adding m-LDH into the PBSA polymer matrix, the composites show an improvement in UV-absorbing character in the whole UV-B and UV-A region. The absorption intensity of PBSA composites increases with increasing m-LDH content. This result indicates that excellent UV absorption property of m-LDH can be used as a UV protecting additive for PBSA. 

**Figure 4:**
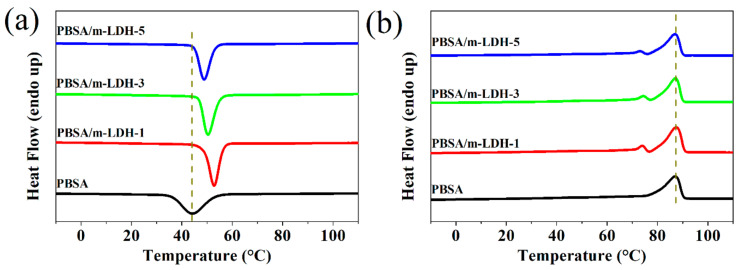
(**a**) DSC 1st cooling and (**b**) 2nd heating curves of PBSA and its composites.

The effect of the chemical modification of nanofiller on polymer composite has been reported by Zhang et al., which shows an increasing T_c_ with modified nanofiller but a decreasing T_c_ by an unmodified nanofiller [33]. In this study, the crystallization behavior of PBSA and its composites were analyzed using 1st cooling and 2nd heating of DSC measurement. The crystallization temperature (T_c_) of PBSA/m-LDH-1 shown in Figure 4a is higher than that of PBSA. With increasing m-LDH content, T_c_ gradually decreased but was still higher than that of PBSA. The detailed data are presented in Table 1. The increase in T_c_ is due to the heterogeneous nucleation caused by m-LDH. Further, increasing m-LDH content may hinder the chain motion of PBSA during crystallization, thus, leading to a decrease in T_c_ [3,20]. 

**Figure A1:**
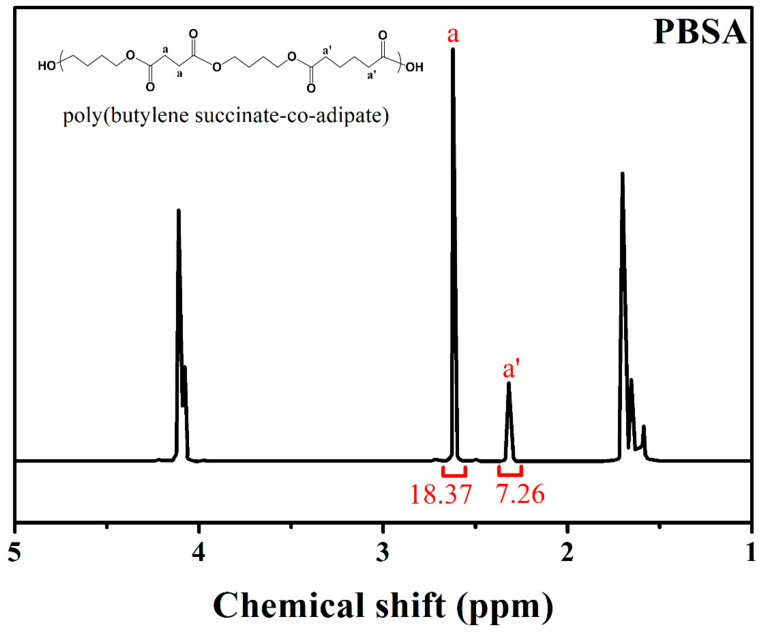
^1^H nuclear magnetic resonance (H-NMR) spectra of PBSA.

Figure 4b shows the melting behavior in the 2nd heating of PBSA and its composites. The melting temperatures (T_m_) of PBSA and its composites are almost the same in all samples, showing that the melting behavior of the PBSA crystallite was not affected by adding fillers. The detailed data are presented in Table 1. An obvious difference observed in the composites with m-LDH revealed a small melting peak appearing before the main peak of samples. These small melting peaks might be attributed to the melt–recrystallization–remelt phenomenon of the PBSA crystallite. The crystallinity of polymer can be calculated by dividing the observed ∆H_f_ by the theoretical value ($\Delta H_{f}^{0}$) for perfectly (100%) crystalline polymer. The theoretical $\Delta H_{f}^{0}$ for polybutylene succinate (PBS) and polybutylene adipate (PBA) are 110.3 and 135.0 J/g, respectively. The theoretical $\Delta H_{f}^{0}$ for PBSA can be calculated via the basis of the butylene succinate (BS)/butylene adipate (BA) group contribution method [34]. In this study, the PBSA material has 72% BS composition (determined via ^1^H nuclear magnetic resonance, shown in Figure A1). The crystallinity of all samples are presented in Table 1. By adding m-LDH into PBSA, the crystallinity increases from 27.4% for PBSA to 31.0, 29.5, and 28.6% for PBSA/m-LDH-1, PBSA/m-LDH-3, and PBSA/m-LDH-5, respectively. The increase in crystallinity might be due to the effect of m-LDH via heterogeneous nucleation. Compared to PBSA, the higher crystallization temperature of PBSA/m-LDH-1 leads to better chain motion during crystal growth at previous cooling process. Higher m-LDH content hinders chain motion during crystallization, which leads to the crystallinity decrease with increasing m-LDH content. At the same time, the lower crystallization temperature compared to PBSA/m-LDH-1 is not conducive to chain motion. Higher m-LDH content hinders chain motion during crystallization, which leads to a decrease in crystallinity with increasing m-LDH content.

### 3.3. Characterization of PBSA/m-LDH Composites after Irradiation

**Figure 5:**
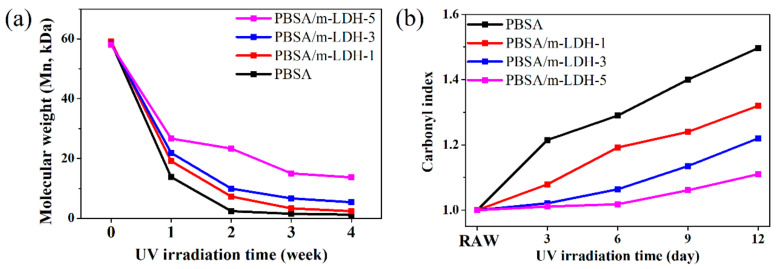
(**a**) Molecular weight (Mn) and (**b**) carbonyl index of PBSA and PBSA/m-LDH composites after different irradiation time.

**Figure A2:**
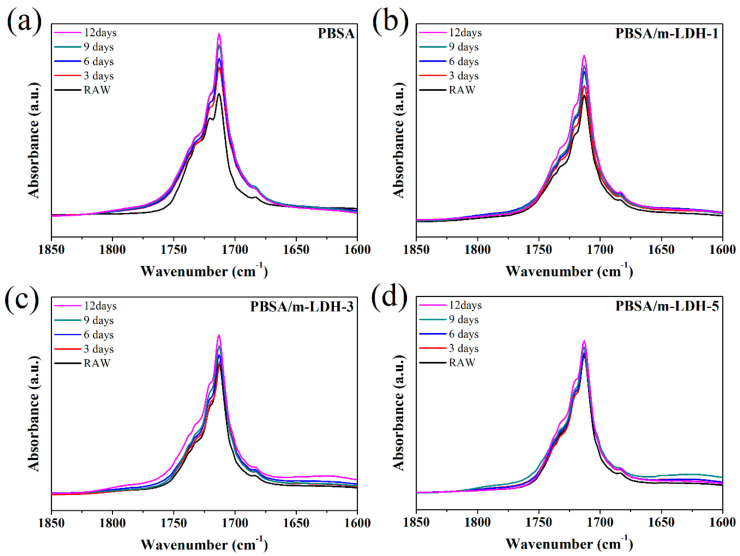
FTIR result of PBSA and its composites after different irradiation time. (**a**) PBSA; (**b**) PBSA-m-LDHD-1; (**c**) PBSA-m-LDHD-3; (**d**) PBSA-m-LDHD-5.

The photodegradation caused by UV could induce the polymer chain scission, which supports the change in molecular weight with increasing irradiation time [14]. Figure 5a shows the change in number average molecular weight (Mn) for PBSA and PBSA/m-LDH composites after a period of irradiation time. These results demonstrate that the photodegradation causes a remarkable reduction in molecular weight in all samples, but the addition of higher m-LDH content could reduce the degradation rates. The result indicates that m-LDH can play a significant role in the photodegradation protection. In addition, the molecular weight of each sample drops sharply in the first week. To understand the difference in the degradation rate of samples on this relatively short irradiation time, the FTIR analysis was applied. As a result of hydroxyl end group oxidation and main chain scission from photolysis at ester linkages, the terminal carboxyl groups are generated. Thus, as irradiation progresses, an increase in C=O peaks is observed in the FTIR spectrum. Therefore, the carbonyl index is defined as [14,29]:

(1) $$\left(\frac{A_{C=O}^{t}}{A_{C-H}^{t}})/(\frac{A_{C=O}^{t0}}{A_{C-H}^{t0}}\right)$$

where $A_{C=O}^{t0}$ and $A_{C=O}^{t}$ are the intensity of carboxyl groups at 1712 cm^−1^ before and after irradiation, respectively; $A_{C-H}^{t0}$ and $A_{C-H}^{t}$ are assigned to the C–H stretching peak at 2858 cm^−1^, which is used as the reference for calculating the value of the carbonyl index, before and after irradiation, respectively [14,28]. Therefore, the higher carbonyl index indicates the poor photostability of materials. The evolution of C=O at 1712 cm^−1^ of PBSA and PBSA/m-LDH composites are shown in Figure A2. Figure 5b shows the carbonyl index of the corresponding samples. The increase of m-LDH content could remarkably reduce the carbonyl index in different irradiation time, which indicates decreased photodegradation of PBSA. 

**Figure 6:**
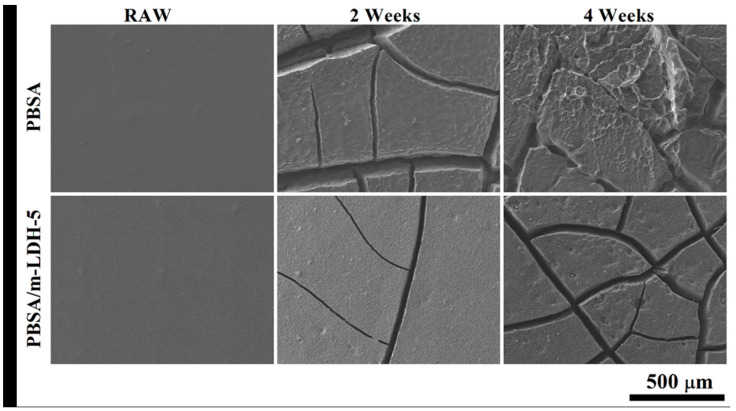
SEM images of surface morphology of PBSA and PBSA/m-LDH-5 after different irradiation time.

The result of samples after artificial photodegradation test shows the m-LDH is an effective nanomaterial to reduce the photodegradation of PBSA. Based on past literature, Zn-Ti LDH has been shown to have UV absorption ability. This study also showed that Zn-Ti LDH keeps this feature after SA modification. The photodegradation of polymer starts from the surface and then develops along the depth [14]. In addition, destruction via photodegradation can induce the entry of oxygen and promote further degradation. For pure PBSA, UV light could enter the inside of the material without additional hindrance, causing the above degradation reaction. For PBSA/m-LDH composites, UV light can be absorbed by m-LDH, which might decreased the photo intensity enter the inside of the material, causing the less degradation reaction. The morphologies of PBSA and PBSA/m-LDH-5 after different UV irradiation time are shown in Figure 6. Prior to irradiation, both of them exhibited a smooth surface with no significant defects. After irradiation, the morphology became rough and was characterized with cracks. After 4 weeks, PBSA showed a rougher surface, indicating a stronger photodegradation behavior than PBSA/m-LDH-5.

## 4. Conclusions

The hydrophobic UV absorption nanofiller was prepared by SA-modified Zn-Ti LDH. The dispersed m-LDH in PBSA matrix shows its stacking structure via WAXD analysis. While not affecting the crystal structure of PBSA, both the crystallization temperature and crystallinity of PBSA/m-LDH composites were increased due to the addition of m-LDH with heterogeneous nucleation. The composite with 1 wt% m-LDH content had the highest T_c_ and crystallinity, which are 52.9 °C and 31.0%, respectively. In addition, the mechanical properties of the composite were enhanced with increasing m-LDH content. By adding 5 wt% m-LDH into PBSA, the storage modulus at −80 °C increased from 4.04 GPa of neat PBSA matrix to 6.16 GPa of composites. The m-LDH decreased the photodegradation behavior of PBSA, which was confirmed by GPC, FTIR, and SEM analysis. This may attribute to m-LDH absorbs the partial incident light entering the composite and reduces the effect of UV radiation. Based on the fact that both Zn-Ti LDH, SA, and PBSA are biocompatible and have low toxicity, the biofriendly composites with photodegradation-resistant properties could be deployed for suitable applications, such as biodegradable mulching films.

## Figures and Tables

**Table 1:** Result of DMA, and DSC analysis for PBSA and its composites.

Sample Name	E′ (Gpa)	E′ (Gpa)	T_c_ (°C)	T_m_ (°C)	ΔH_f_ (J/g)	X_c_ (%)
PBSA	4.04	0.43	44.3	87.1	32.1	27.4
PBSA/m-LDH-1	4.42	0.46	52.9	74.0/87.4	36.3	31
PBSA/m-LDH-3	5.19	0.5	50.4	74.3/86.9	34.6	29.5
PBSA/m-LDH-5	6.16	0.63	48.8	73.3/87.1	33.5	28.6

${E\prime }_{-80}$ and ${E\prime }_{25}$ : storage modulus at −80 and 25 °C, respectively, measurement by DMA. T_c_: crystallization temperature during 1st cooling trace, measurement by DSC. T_m_: crystalline melting temperature during 2nd heating trace, measurement by DSC. X_c_: crystallinity, which obtained by the following equation, $\mathrm{Xc}\left(\%\right)=\Delta H_{f}/\left[\left(1-\varphi \right)\Delta H_{f}^{0}\right]\times 100\%$, where $\Delta H_{f}^{0}$ = 117.2 Jg^−1^ for PBSA with 72% BS group ratio, and φ is the weight fraction of the filler in the composites.

## Data Availability

Not applicable.

## Appendix A

**Table A1:** Results of TGA analysis for Zn-Ti LDH, m-LDH and stearic acid.

Sample Name	10 wt% Loss Temperature (°C)	Char Yield at 600 °C (wt%)
Zn-Ti LDH	269.2	75.0
m-LDH	292.0	46.9
stearic acid	218.7	0.5
